# Comparison of population-genetic structuring in congeneric kelp- versus rock-associated snails: a test of a dispersal-by-rafting hypothesis

**DOI:** 10.1002/ece3.16

**Published:** 2011-10

**Authors:** Raisa Nikula, Hamish G Spencer, Jonathan M Waters

**Affiliations:** Allan Wilson Centre for Molecular Ecology and Evolution, Department of Zoology, University of OtagoP.O. Box 56, 9016 Dunedin, New Zealand

**Keywords:** Connectivity, *Diloma*, *Durvillaea antarctica*, habitat, Macroalga, microsatellites

## Abstract

Phylogeographic studies indicate that many marine invertebrates lacking autonomous dispersal ability are able to achieve trans-oceanic colonization by rafting on buoyant macroalgae. However, less is known about the impact of rafting on on-going population-genetic connectivity of intertidal species associated with buoyant macroalgae. We hypothesize that such species will have higher levels of population-genetic connectivity than those exploiting nonbuoyant substrates such as rock. We tested this hypothesis by comparing nuclear multilocus population-genetic structuring in two sister topshell species, which both have a planktonic larval phase but are fairly well segregated by their habitat preference of low-tidal bull-kelp holdfasts versus mid-to-low tidal bare rock. We analyzed population samples from four sympatric sites spanning 372 km of the east coast of southern New Zealand. The sampled region encompasses a 180 km wide habitat discontinuity and is influenced by a stable, northward coastal current. The level of connectivity was high in both species, and neither of them showed significant correlation between genetic and geographic distances. However, a significant negative partial correlation between genetic distance and habitat discontinuity was found in the rock-associated species, and estimates of migrant movement between sites were somewhat different between the two species, with the kelp-associated species more often yielding higher estimates across the habitat discontinuity, whereas the rock-associated species more often exhibited higher estimates between sites interspersed by rock habitats. We conclude that for species with substantial means of autonomous dispersal, the most conspicuous consequence of kelp dwelling may be enhanced long-distance dispersal across habitat discontinuities rather than a general increase of gene flow.

## Introduction

Population connectivity or exchange of genetic material between geographical populations is a key topic in marine ecology, with a wealth of studies having addressed the role of planktonic larvae in facilitating gene flow in otherwise nondispersive species (reviewed by Kinlan and Gaines 2003; Cowen and Sponaugle 2009). Very few studies, however, have addressed the evolutionary importance of marine dispersal mechanisms that are linked with habitat association during postlarval life stages. Intertidal ecosystems of the world's cool and temperate regions are characterized by large, often buoyant, perennial macroalgae that support substantial levels of marine biodiversity. Whereas some of the invertebrate species associated with such macroalgal “forests” (kelp beds) are highly mobile and exploit many parts of these three-dimensional habitats, many taxa are sessile as adults and rely on planktonic larvae for dispersal beyond their home site. The common ecological association of sedentary marine animals with large buoyant macroalgae may have consequences for their population-genetic connectivity at various spatial scales. Empirical evidence from phylogenetic studies of macroalgal-associated species suggests that some taxa associated with buoyant macroalgae have been able to disperse well beyond their autonomous dispersal range over fairly recent (Holocene) times (Donald et al. 2005; Nikula et al. 2010). These observations imply that epifauna associated with buoyant macroalgae could have, owing to passive rafting, greater potential for dispersal and gene flow than species inhabiting rocky intertidal habitats and/or nonbuoyant macroalgae.

**Figure 1:**
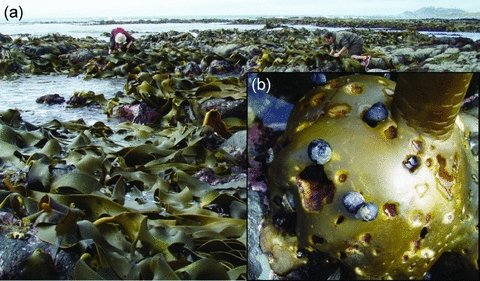
(A) Southern bull-kelp (*Durvillaea antarctica*) beds at low tide near Kaka Point, New Zealand; (B) *Diloma* snails grazing on the surface of a bull-kelp holdfast. Photos courtesy of CI Fraser.

The large brown seaweed orders Fucales and Laminariales contain numerous buoyant taxa, including *Durvillaea antarctica* (Fig. 1A), *Fucus vesiculosus*, *Macrocystis pyrifera*, and *Ecklonia radiata* (Raven et al. 2005). When such buoyant seaweeds become detached from their rocky substrate by wave action, they and their epifauna may get transported over long distances to new locations by currents and winds (Fraser et al. 2011). Low levels of population-genetic differentiation within several buoyant macroalgal species have been attributed to gene flow mediated by drifting but still reproductively active adults (Coleman and Brawley 2005; Muhlin et al. 2008; Coleman and Kelaher 2009; Fraser et al. 2010). This genetic evidence for transportation of whole adult specimens between macroalgal stands suggests that drifting of macroalgae may also have potential to shape population-genetic structuring of “hitchhikers” (i.e., associated epifaunal species).

Rafting is often invoked *post hoc* to explain unexpectedly high levels of long-distance population-genetic connectivity in marine coastal species (e.g., Bell 2008; McCormick et al. 2008; Leese et al. 2010). Nevertheless, rigorous tests of the evolutionary effects of rafting on dispersal and gene flow of animal species associated with macroalgae are generally lacking. To control for the effects of other dispersal mechanisms, such tests would ideally involve contrasting population-genetic structures of codistributed species that share life-history characteristics relevant for autonomous dispersal ability, but are differentially prone to rafting on macroalgae. In this study, we explore the effects of macroalgal rafting on genetic connectivity of intertidal invertebrates by studying multilocus population-genetic structuring of two New Zealand co-distributed, phylogenetically sister, intertidal mollusc species from the genus *Diloma* (Gastropoda: Trochidae: Monodontinae). While a recent genetic study compared large-scale genetic connectivity in two other *Diloma* species, one of which is specialized on feeding on decaying fragments of bull-kelp in high intertidal and one of which inhabits mudflats (Donald et al. 2011), the current study is the first to analyze a sister species pair in sympatry to quantify the impact of habitat association on population-genetic connectivity.

The topshells *Diloma arida* (Finlay 1926) and *D. durvillaea* (Spencer et al. 2009) are morphologically cryptic species, whose speciation is suggested to have taken place as a result of habitat specialization (Spencer et al. 2009). *Diloma arida* lives throughout coastal New Zealand in shallow rock pools and rocky surfaces immediately above the low intertidal bull-kelp zone and up into the mid-intertidal zone, whereas *D. durvillaea* is typically found inhabiting holdfasts of the southern bull-kelp *D. antarctica* (Cham.) or, occasionally, crawling on rock surfaces nearby (Spencer et al. 2009; Fig. 1B). These separate species were unrecognized until detailed conchological inspections of a large number of individuals found from inside bull-kelp holdfasts and subsequent phylogenetic analyses of mitochondrial DNA prompted the taxonomic description of *D. durvillaea* (Spencer et al. 2009). *Diloma durvillaea* apparently has a more restricted distribution than *D. arida*: it has so far been found only from scattered locations south of Banks Peninsula on the east coast of mainland New Zealand and in the subantarctic Auckland Islands (Spencer et al. 2009). Genetic studies of beach-cast *D. antarctica* (Collins et al. 2010) and some of its epifaunal invertebrates (Fraser et al. 2011) indicate that transportation of bull-kelp rafts takes place at a spatial scale that easily encompasses the whole area where these two *Diloma* species are known to coexist.

Species of the genus *Diloma* reproduce by broadcasting their eggs and sperm freely into the water column (Grange 1976). While the mode of larval feeding and duration of the larval period have not been specifically studied in the genus, studies of many other trochid gastropods (Hickman 1992), especially of the Monodontinae (Underwood 1974), suggest that the larval phase is almost certainly lecithotrophic and lasts up to 1 week (cf. Donald et al. 2011). Furthermore, as both species occupy adjacent intertidal habitats that are submerged at high tide when broadcast spawning takes place, we postulate that the larval dispersal potential of these two species is similar. Differences between the species that could impact on population connectivity are their different distribution patterns and population sizes: the rock-associated *D. arida* is much more abundant and continuously distributed along the east coast of New Zealand than *D. durvillaea*. Assuming these differences have minimal effect, we can potentially ascribe any major population-genetic differences between these two taxa to the effects of rafting dispersal of postsettlement *D. durvillaea* on detached bull-kelp.

More specifically, if effective dispersal by rafting takes place in *D. durvillaea*—in addition to larval dispersal that both *D. durvillaea* and *D. arida* undergo—we expect *D. durvillaea* to exhibit more frequent migration and hence lower genetic differentiation and genetic distances between populations than *D. arida* (Hypothesis 1). Given that bull-kelp beds, the primary habitat of *D. durvillaea*, may undergo intermittent local extinction and regeneration cycles (Donald et al. 2011) and also have a patchier distribution than the rocky intertidal surfaces where *D. arida* occurs, frequent long-distance dispersal by rafting may also result in highly unpredictable recruitment patterns of *D. durvillaea*, and thus to weak correlation between geographic distance and genetic differentiation of populations (Hypothesis 2).

## Materials and Methods

### Study area and sample collection

**Figure 2:**
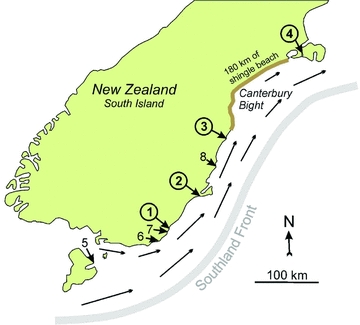
Sampling sites of *Diloma arida* and *D. durvillaea* in the east coast of New Zealand. Samples of both species were analyzed for genetic connectivity from the circled sites; only *D. arida* samples were analyzed from the other sites. Location of the Southland Front bounding the Southland Current (direction indicated by arrows) was drawn after Chiswell (1996).

The large-scale oceanography of our study area in the south-eastern coast of New Zealand is characterized by a major north-east flowing Southland Current that is generated by the Southland Front, at the interface between subtropical and subantarctic surface waters (Fig. 2). A 180-km-long stretch of alluvial mixed sand and gravel beach, devoid of any stable intertidal rocks that are necessary for the establishment of bull-kelp beds, is located in the northern part of the study area along the Canterbury Bight (Eikaas and Hemmingsen 2006) and separates the northern-most collection site (no. 4) from those further south.

We collected samples of rock-dwelling and bull-kelp-dwelling *Diloma* from eight and five sites, respectively (Fig. 2; Table 1), at low tide (Fig. 1A). Specifically, we obtained sufficiently large samples of *Diloma* from both habitats from four sites (nos. 1–4) that spanned a total 372 km of coastline: these samples provided the basis for between-species comparisons. We also collected rock-dwelling *Diloma* from four additional sites, to provide insight into geographic structuring of *D. arida* across distances up to 516 km. In spite of searching, we did not find holdfast cavity-dwelling *Diloma* in these four localities, but did collect individuals that were grazing on the outer surface of holdfasts and fronds. We collected the kelp-associated *Diloma* (presumed *D. durvillaea*) from holdfast cavities of *D. antarctica* by detaching the holdfasts from their rocky substrate using an axe and/or a wrench bar. We collected rock-associated *Diloma* (presumed *D. arida*) by hand from rock surfaces and shallow rock pools in the vicinity of bull-kelp beds. We immersed the animals in 96% ethanol upon collection, with samples from each habitat stored and labeled separately. We replaced the ethanol after 1–2 days, and stored the samples at 4°C in the laboratory prior to analyses.

**Table 1:** Sampling sites, their geographic coordinates, and sample sizes of *Diloma arida* and *D. durvillaea* at each site from intertidal rock- and bull-kelp habitats. Species identity of each individual was determined by genetic cluster assignment. Samples in parentheses were not used in the analyses of population connectivity due to their insufficient size

		*Diloma arida*		*Diloma durvillaea*	
			
Site number and locality name	Lat, long coordinates	Rock	Kelp	Rock	Kelp
1. North of Kaka Point, Southwest of Dunedin	46° 22.890′S; 169° 47.017′E	30	1	2	16
2. St Clair, Dunedin	45° 54.858′S; 170° 29.317′E	31	0	8	35
3. All Day Bay, Southwest of Oamaru	45° 11.707′S; 170° 53.850′E	21	1	5	23
4. Te Oka Bay, Banks Peninsula	43° 51.205′S; 172° 47.017′E	23	0	4	31
		Subtotal 107		Total 124	
5. Ringaringa, Stewart Island	46° 54.115′ S; 168° 08.803′ E	18	3^1^	(2)	(1^1^)
6. Jacks Bay, South of Balclutha	46° 29.947′ S; 169° 42.618′ E	28	0	(2)	(0)
7. South of Kaka Point, Southwest of Dunedin	46° 24.127′ S; 169° 47.185′ E	24	17^1^	(1)	(2^1^)
8. Shag Point, Northeast of Dunedin	45° 27.930′ S; 170° 49.650′ E	24	0	(2)	(6 + 2^1^)
		Total 221			

1 found and collected not from holdfast cavities like the other kelp-associated individuals, but from outer surface of bull-kelp.

Literature suggests that the habitats of these two species are well segregated, with rock-associated *D. arida* having never been encountered inside *D. antarctica* holdfasts (Spencer et al. 2009). The holdfast-associated *D. durvillaea*, on the other hand, has occasionally been found to occupy rock surfaces adjacent to bull-kelp beds (The Museum of New Zealand Te Papa Tongarewa voucher specimens, coll. W. R. B. Oliver 1920; H.G. Spencer, pers. obs.). Although our observations during field collections were generally consistent with this reported ecological segregation, we did find some instances of ecological overlap: specimens with morphological features characteristic of *D. arida* were occasionally found on the outside of holdfasts and on fronds of *D. antarctica*. In addition, despite the subtle qualitative differences in adult shell features, we note that the two species are not known to have readily quantifiable, diagnostic morphological characteristics, and that subadult individuals can be especially difficult to distinguish (Spencer et al. 2009; B. A. Marshall, pers. comm.). To discover if any of the analyzed specimens came from atypical habitat (i.e., *D. arida* from bull-kelp holdfasts/*D. durvillaea* from rock surfaces), and to avoid erroneous inclusion of *D. durvillaea* specimens in the *D. arida* dataset and vice versa, we initially performed a genetic assignment analysis on the whole two-species dataset, assuming that two noninterbreeding clusters are present in the data, and then used the cluster membership estimate of each specimen to assign its species identity (see Data analyses for details).

### Microsatellite marker development

The microsatellite markers for this study were developed by constructing an enriched genomic library of *D. durvillaea* for AG, TG, AAC, AAG, ACT, and ATC repeats following Glenn and Schable (2005). We screened the library for microsatellite-positive clones following Perrin and Roy (2000). From the total of 697 clones, we amplified and sequenced 39 clearly positive clones with M13-primers using the BigDye Cycle Sequencing Kit and an ABI 3730 Genetic Analyser (Applied Biosystems, Foster City, CA, USA). We found eleven of the sequenced clones to contain microsatellite repeats that were shorter than 180 base pairs and therefore suitable for visualization on 16 cm long, 8–10% acrylamide gels. We designed primer pairs for PCR amplification of these microsatellite regions with Primer3Plus (Untergasser et al. 2007). We checked the loci for the presence of allelic polymorphism by first analyzing six specimens of both species from St Clair, Dunedin. We optimized PCR amplification conditions by working on DNA of approximately 50 specimens of each species across all sample localities. After genotyping more specimens, we found four of the loci to have a very high amplification-failure rate and discarded them. The final marker set consisted of seven loci; their primer sequences, repeat motifs, amplification conditions, diversity statistics, and GenBank accession numbers of the respective cloned sequences are presented in Table S1 and were submitted to the Molecular Ecology Resources Primer Database.

### Microsatellite genotyping

We extracted DNA from the topshells using either Chelex (Walsh et al. 1991) or an alternative method described by Zavodna et al. (2008). We found the latter method to yield better-quality DNA template for microsatellite amplification, and the majority of extractions were subsequently performed using this method. We extracted DNA from a piece of tissue dissected from under the operculum, a body region we expected to be well preserved due to its immediate contact with ethanol. Upon extraction, we retained the dried shell and ethanol-preserved soft tissue of every study individual; they were deposited in collections of The Museum of New Zealand Te Papa Tongarewa in Wellington.

We amplified DNA of the topshells at seven microsatellite loci in separate 10-µl volume PCR reactions. PCR mixture contained 1–4 µl of DNA extraction solution, 0.5 µM of each primer, 0.75 U Mango *Taq* DNA polymerase (Bioline, London, United Kingdom), 1×*Taq* buffer (Bioline), 0.8 µM dNTP, 2.0 mM MgCl_2_ and 1 µg BSA (Invitrogen, Carlsbad, CA, USA). We resolved the amplified DNA fragments on 8–10%, vertical acrylamide gels using the Hoefer™ SE 600 Chroma equipment (Hoefer Inc., San Francisco, CA, USA), in 10–11 mA current. We ran 10-base pair DNA ladder (Invitrogen) on the edge and middle lanes of each gel to size the microsatellite fragments. We stained electrophoresed gels in SYBR Green (Invitrogen) solution to visualize the fragments, photographed them with GelDoc 2000 (Uvitec, Cambrigde, United Kingdom), and later scored the alleles from digital and printed photographs without information about the population or habitat origin of the individual being scored. We controlled the consistency of our allele scoring by rerunning some previously scored, heterozygous samples alongside newly genotyped samples. Amplification and scoring success varied between individuals and loci; we restricted the final sample set and analyses to individuals that were successfully genotyped at four or more loci. Amplification of the DNA samples at more than half of the loci and concurrent amplification failure at some loci despite several PCR trials suggests that the missing genotypes were largely a result of null alleles (i.e., mismatch of the primers with flanking regions). Therefore, we adjusted our analyses for presence of null alleles whenever possible. The genotype data used in the analyses, complete with habitat- and museum specimen voucher information, were deposited in the Dryad data repository (http://datadryad.org/).

### Data analyses

Initially, we analyzed the total 7-locus dataset, consisting of 413 individuals of the two morphologically cryptic sister species, with the individual-based MCMC-clustering analysis in the STRUCTURE software (Pritchard et al. 2000) in order to verify/determine the species identity of each individual. Assuming that our dataset consists of two biological species, we determined the membership proportions (*Q*_1_, *Q*_2_) of each individual in two hypothetical populations (*K*= 2) under the model of nonadmixture and independent allele frequencies. These analyses ignored our prior expectations about species identity based on ecology and/or morphology. We started the simulations with a burn-in period of 50,000 iterations, followed by 200,000 MCMC iterations. We ensured the convergence of the MCMC chains by running five independent iterations of each simulation that yielded virtually identical results. We deemed individuals receiving a >80% membership estimate (*Q*-value) for either of the two hypothetical populations to qualify for further analyses as representing *D. arida*/*D. durvillaea* and discarded individuals with *Q*-values between 0.2 and 0.8 from all subsequent analyses.

We obtained basic descriptive statistics of the datasets, such as total allele numbers and allele size ranges per locus, using GenAlEx v. 6.4.1 (Peakall and Smouse 2006). We used MICRO-CHECKER v. 2.2.3 (Van Oosterhout et al. 2004) to test the loci for Hardy-Weinberg equilibrium (HWE) within samples, and to assess whether homozygote excesses in the HWE-deviant samples were most likely due to technical artifacts (e.g., null alleles or large allele drop-out) or nonrandom interbreeding (e.g., Wahlund-effect). Because of evidence of null alleles at every locus, we used FreeNA (Chapuis and Estoup 2007) to estimate corrected allele frequencies that accounted for their presence. We then analyzed these allele frequencies with the software DISPAN (Ota 1993) to obtain estimates of expected heterozygosity (*H*_S_) within sample populations, and total heterozygosity (*H*_T_) that took into account the estimated frequencies of null alleles in sample populations. We estimated *H*_S_ and *H*_T_ across the total four-population sample of each species and from all population pairs within species.

We measured the global genetic structuring of the species with three types of statistics—*D*_est_, *F*_ST_, and *G*″_ST_—that complement each other regarding different viewpoints to population structuring, that is population differentiation versus gene flow or migration between populations (Meirmans and Hedrick 2011). We quantified global genetic differentiation among the four sample localities in each species by estimating mean *D*_est_ (Jost 2008) over loci, using the package DEMEtics version 0.8 (Gerlach et al. 2010) in R version 2.11.1 (R Development Core Team 2010). *D*_est_ is more appropriate measure of population differentiation than *F*_ST_ with markers that have high allelic diversity, as its maximum value is not limited by the within-population heterozygosity as is that of *F*_ST_ (Jost 2008; Gerlach et al. 2010; Meirmans and Hedrick 2011). We generated 1000 random bootstrap data resamplings to obtain 95% confidence intervals for mean *D*_est_ estimates. Either alleles or genotypes were resampled, depending on whether the contrasted populations were in HWE at a locus or not, respectively. To enable estimation of global and between-locality migrant numbers (*N*_m_) unaffected by within-population heterozygosity as per Meirmans and Hedrick (2011), we calculated global and population-pairwise values of the fixation indices *F*_ST_ and *G*″_ST_ (Meirmans and Hedrick 2011). For calculation of *F*_ST_, we used FreeNA (Chapuis and Estoup 2007), which provides estimates corrected for the presence of null alleles, and uncorrected estimates calculated following Weir (1996). We calculated global and population-pairwise *G*″_ST_ estimates according to Meirmans and Hedrick's (2011) Equation 4:

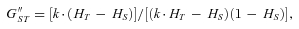
where total heterozygosity (*H*_T_) and expected heterozygosity within sample populations (*H*_S_) were average values over the seven loci, obtained with DISPAN as explained above; *k* is the number of sampled populations (*k*= 4 in case of global analyses, and *k*= 2 in case of population-pairwise analyses). The estimate of the number of migrants per generation (*N*_m_) under the equilibrium island model assumption was then calculated following Meirmans and Hedrick (2011) for both species globally and for all population pairs within each species as *N_m_*= (1 –*G*″_ST_)/4*F*_ST_.

We used FreeNA also for calculation of population-pairwise Cavalli-Sforza and Edwards’ (1967) distances (*D*_C_ distances) corrected for the presence of null alleles. We then analyzed the correlations and partial correlations of population-pairwise *D*_C_ distances, geographic distances, and major habitat discontinuity (180-km habitat gap in the Canterbury Bight) by conducting Mantel tests in Isolation By Distance Web Service (Jensen et al. 2005). Statistical significance levels of the correlations and partial correlations (controlled for one of the variables) were tested against 30,000 random bootstrap replicates. We obtained the geographic distances between the sampling localities by measuring the shortest marine route between each locality pair in Google Earth.

We also applied a fourth analysis method that was recently developed for quantification of subtle geographic structuring especially in species with high gene flow: spatial analysis of shared alleles (SAShA) that looks at mean distance between co-occurrences of an allele in geographically spread data (Kelly et al. 2010). We expected to find a larger observed mean distance (OM) in *D. durvillaea* than in *D. arida* owing to the presumed more frequent rafting potential of the former. In addition to comparing the mean values of the statistic in the two species, permitted by completely overlapping field sampling regime across four sampling localities, we tested those datasets and also the eight-population *D. arida* data for the presence of significant geographic structuring of allele occurrences against 10,000 random bootstrap replicates that provided null distributions expected under panmictic conditions.

## Results

### Species determination with genetic cluster assignment analysis

We found the expected strong match between multilocus genotype cluster and habitat (i.e., *D. durvillaea* in/on bull-kelp versus *D. arida* on rock) for 76% of the genotyped individuals (315 out of 413). However, 13% (or 48) of the individuals were strongly assigned to the genetic cluster that was unexpected based on their collection habitat (Table 1). Twenty-six individuals collected from rock surfaces genetically resembled (mean *Q*_1_= 0.94, *SD*= 0.066) those collected from inside bull-kelp holdfasts that were preliminarily identified as *D. durvillaea* based on shell characteristics. In contrast, only two individuals collected from inside bull-kelp holdfasts (at sites nos. 1 and 3) were assigned strongly (*Q*_2_ > 0.92) to the “*D. arida*” genetic cluster, members of which were predominantly collected from rock surfaces and identified morphologically as *D. arida*. In addition, 20 individuals collected from top of bull-kelp holdfasts or fronds at sites nos. 5 and 7 grouped genetically (mean *Q*_2_= 0.98, *SD*= 0.023) with the predominantly rock-dwelling cluster and were subsequently regarded as *D. arida*. Five individuals collected from holdfast outer surfaces associated strongly (*Q*_1_ > 0.98) with the “*D. durvillaea*” cluster. Eighteen of the “*D. durvillaea*” cluster genotypes were not included in further analyses because they came from localities where too few “*D. durvillaea*” had been found to warrant meaningful analyses (see Table 1). We note that *D. arida* was found in numbers on bull-kelp only in a locality where *D. durvillaea* was absent from holdfast cavities at the time of sampling, and was absent from bull-kelp surfaces in localities where *D. durvillaea* was found in holdfast cavities. In summary, our genetic data suggest that when sympatric, the frequency at which *D. durvillaea* occurs on rock substrate is far higher than the frequency at which *D. arida* exploits bull-kelp substrate, and that *D. arida* only very rarely utilizes the cavities of bull-kelp holdfasts (Tables 1 and 4).

**Table 4:** Habitat segregation and global statistics on population-genetic connectivity in *D. arida* and *D. durvillaea*, estimated based on microsatellite data at seven loci over population samples from sites nos. 1–4. CI—confidence interval from bootstrap-replicating over loci. ENA—estimate obtained using null allele frequency estimation. *N*_m_—estimated number of migrants exchanged between subpopulations per generation

	*D. durvillaea*	*D. arida*
Proportion of the sampled individuals that inhabited bull-kelp holdfast cavities	85%	2%
Within-population heterozygosity *H*_S(ENA)_, averaged over loci	0.732	0.737
Total heterozygosity *H*_T(ENA)_, averaged over loci	0.749	0.755
Mean *D*_est_ (95% CI)	0.090 (0.044, 0.135)	0.083 (0.042, 0.124)
*F*_ST_ (95% CI)	0.004 (–0.003, 0.018)	0.006 (–0.005, 0.026)
*F*_ST(ENA)_ (95% CI)	0.011 (–0.001, 0.033)	0.007 (–0.002, 0.025)
*G*″_ST(ENA)_	0.113	0.118
*N*_m_, as inferred from (1–*G*″_ST_)/4*F*_ST(ENA)_	20.2	31.5
Observed mean distance (OM) between co-occurrences of alleles, km	148.1	143.4
Expected mean distance (EM) between co-occurrences of alleles under panmixia, km	150.0	146.6
*P*-value for the null hypothesis OM = EM, from random permutation test	*P*= 0.43	*P*= 0.25

The remaining 12% of the samples—37 individuals collected from rock surfaces, three from the blades of bull-kelp, and 10 collected from inside bull-kelp holdfasts—were found to be genetically “intermediate” (0.20 < *Q*_i_ < 0.80, *SD*= 0.19) and were omitted from further analyses due to the apparent uncertainty of their species identity. Most likely their intermediate *Q*-values were due to data missing at the loci that best diagnose *D. arida* and *D. durvillaea*: 39 of the “intermediate” individuals were missing genotypes on at least one of the top three between-species differentiated loci (Ddu5-Ddu7) and in general had a higher proportion of missing data (23%) than the individuals that received *Q*-values above 0.8 (14%). An alternative explanation is that these individuals are interspecific hybrids, although the present dataset is not sufficient to resolve this issue. Based on the morphological examination of the genetically intermediate specimens by B. A. Marshall at The Museum of New Zealand Te Papa Tongarewa, hybrid status is not out of the question.

### Polymorphism and null alleles

Among the total 363 study individuals with clear species assignation (listed in Table 1), genotypic data were more complete in the genetic cluster determined as *D. durvillaea* (91% of single-locus genotypes scored) than in *D. arida* (83%). Amplification of the locus Ddu7 was particularly unpredictable in *D. arida*, failing in 56% of the individuals that otherwise amplified at four or more loci, while its failure rate in *D. durvillaea* was only 16%. Ddu7 was nevertheless included in the analyses, because it showed a consistent, albeit low, scoring rate across the *D. arida* localities.

Allelic polymorphism at the seven microsatellite loci was high in both species, with the number of alleles detected per locus ranging between five and 41 in *D. durvillaea* and between five and 39 in *D. arida* (Table 2). Most of the high-frequency alleles (frequency > 0.05) were shared between species, but high-frequency, species-specific alleles were detected at all loci except Ddu3. Alleles at the most polymorphic loci (Ddu2, Ddu4, and Ddu7) were typically rare (frequency < 0.05) and private to one species.

**Table 2:** Allele size range, number of alleles detected, and range of estimated null allele frequencies at seven microsatellite loci over population samples from sites nos. 1–4 of *D. arida* and *D. durvillaea*

	Allele size range (bp)		Number of alleles		Null allele frequency	
			
Locus	*D. arida*	*D. durvillaea*	*D. arida*	*D. durvillaea*	*D. arida*	*D. durvillaea*
Ddu1	76–280	82–280	15	17	0.08–0.23	0.17–0.22
Ddu2	100–270	112–255	37	38	0.09–0.17	0.19–0.30
Ddu3	155–220	133–252	5	13	0.09–0.26	0–0.24
Ddu4	108–300	126–285	39	37	0.19–0.22	0.10–0.19
Ddu5	65–122	65–140	9	16	0.08–0.23	0.05–0.09
Ddu6	130–152	133–140	7	5	0–0.13	0–0.15
Ddu7	126–255	115–197	41	21	0.26–0.30	0.19–0.21

We observed significant excesses of homozygotes at most loci and in most of the sample populations. Nevertheless, loci Ddu3, Ddu4, and Ddu5 were usually at HWE in *D. durvillaea* samples, and loci Ddu5 and Ddu10 were at HWE in *D. arida*. MICROCHECKER analyses revealed that observations of homozygote excess were always compatible with the patterns expected from presence of null alleles in the data, and incompatible with patterns expected from Wahlund-effects or allele “drop-out.” Null alleles were estimated to be present at high frequencies in populations of both species: a summary of null allele frequency estimates per sample and per species is presented in Table 3. Estimates of null allele frequencies per locus in each species are shown in Table 2.

**Table 3:** Average estimates and standard deviations (in parentheses) of null allele frequencies over seven microsatellite loci in population samples from sites nos. 1–4 of *D. arida* and *D. durvillaea*

Site no.	*D. arida*	*D. durvillaea*
1.	0.171 (0.044)	0.150 (0.093)
2.	0.151 (0.099)	0.148 (0.097)
3.	0.115 (0.097)	0.192 (0.044)
4.	0.158 (0.073)	0.180 (0.07)

### Subtle genetic structuring but distinct geographical patterns of connectivity

**Figure 3:**
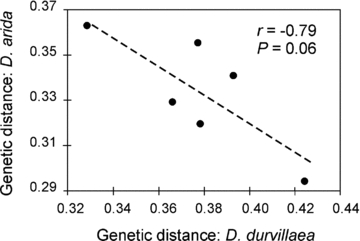
Population-pairwise *D*_C_ distances of *D. durvillaea* plotted against the spatially coinciding distances of *D. arida*. Pearson correlation coefficient (*r*) and its two-tailed *P*-value are shown; dashed line marks the fitted linear correlation.

*Diloma arida* and *D. durvillaea* both exhibited very low levels of population structuring overall (Table 4). According to *D*_est_ values and their 95% confidence intervals, both species showed low but significant population-genetic differentiation. Global *F*_ST_ estimates were not significantly different from zero in either species, but this result was expected given their high within-population allelic diversity. We did not find significant differences in the strength of the overall structuring between the two species as measured by mean *D*_est_ and *F*_ST_, corrected or uncorrected for null alleles, and their 95% confidence intervals. The inferred mean number of migrants between populations was 1.5 times larger in *D. arida* than in *D. durvillaea*, but the overlapping confidence intervals of the *F*_ST_ estimates (central for point estimation of *N*_m_) indicate this difference is nonsignificant. Also, SAShA suggests that both species are panmictic over the area of overlapping sampling, even though *D. durvillaea* has approximately 5 km wider mean distance between co-occurrence of shared alleles than *D. arida*. Summed over all population pairs, the *D*_C_ distances were slightly, but not significantly, higher in the kelp-associated *D. durvillaea* (mean: 0.378; *SD*= 0.032) than in the rock-associated *D. arida* (mean: 0.334; *SD*= 0.025). However, the population-pairwise *D*_C_ distances of the species were strongly negatively correlated; the statistical significance level of the negative correlation was 6% (Pearson's correlation coefficient *r*=–0.073, two-tailed *P*= 0.06; Fig. 3).

**Figure 4:**
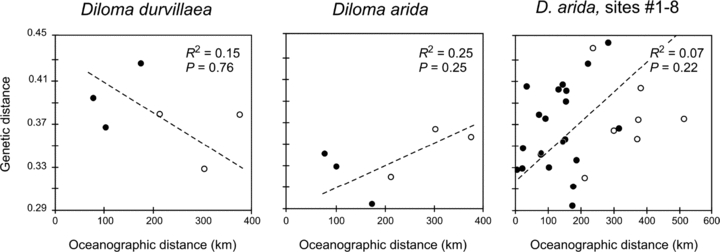
Pairwise *D*_C_ distances of four codistributed *D. durvillaea* and *D. arida* populations, and of eight *D. arida* populations, plotted against geographic distance between the sampling sites. Closed circles—site pairs connected by rocky intertidal habitat; open circles—site pairs separated by a 180-km habitat discontinuity at the Canterbury Bight. Dashed line—reduced major axis regression for the total correlation of genetic and geographic distances, uncontrolled for habitat discontinuity. Global and partial correlations and their Mantel test results are reported in Table 5.

**Figure 5:**
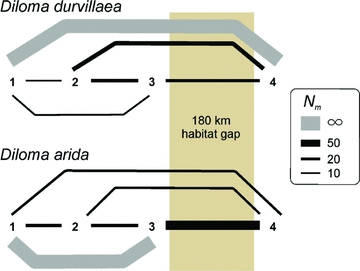
Estimates of migrant numbers (*N*_m_) exchanged between populations of *D. arida* and *D. durvillaea* during a generation, calculated as *N*_m_= (1–*G*″_ST[ENA]_)/(4*F*_ST[ENA]_). ∞—indefinite, owing to *F*_ST_≤ 0.

We detected some differing trends between the species in the geographic patterns of genetic differentiation. Specifically, rock-associated *D. arida* showed a strongly positive and statistically highly significant correlation of geographic distances and genetic *D*_C_ distances when the effect of habitat discontinuity was removed (i.e., controlled for; Table 5). In contrast, the kelp-associated *D. durvillaea* showed a negative but statistically nonsignificant trend between genetic and geographic distances, and that correlation remained nonsignificant even after removal of the effect of habitat discontinuity (Fig. 4; Table 5). Similarly, the estimates of migrant numbers between localities yielded very different patterns between the two species. When summed over all pairwise comparisons across the Canterbury Bight, the kelp-associated *D. durvillaea* seems to have exchanged more migrants across the habitat gap than the rock-associated *D. arida* (Fig. 5). Moreover, we inferred the highest migrant numbers in *D. durvillaea* to have moved between the localities furthest apart (between sites nos. 1 and 4). By contrast, we inferred the rock-associated species to have exchanged more migrants than the kelp-associated species in two of three comparisons between the sites south of the gap, in an area where rocky intertidal habitat is never uninterrupted by sandy or gravel beaches more than 8 km long, and also between sites nos. 3 and 4, the closest site pair separated by the habitat gap (Fig. 5).

**Table 5:** Coefficients of correlation and partial correlation between Cavalli-Sforza and Edwards’ genetic distances corrected for null alleles (Gen), geographic distance (Geo), and major habitat discontinuity (Gap) between population samples of *D. durvillaea* and *D. arida*, and their statistical significance level from Mantel test (one-sided *P*-value). Pairwise comparisons across the Canterbury Bight (see Fig. 2) were classified as involving a major habitat discontinuity. The results on the first row are presented graphically in Figure 3

Variables	*D. durvillaea* sites nos. 1–4	*D. arida* sites nos. 1–4	*D. arida* sites nos. 1–8
Gen, Geo	−0.39 (*P*= 0.76)	0.50 (*P*= 0.25)	0.27 (*P*= 0.22)
Gen, Geo; controlled for Gap	−0.53 (*P*= 0.76)	0.94 (*P* < 0.00)	0.27 (*P*= 0.21)
Gen, Gap	0.51 (*P*= 0.33)	−0.77 (*P*= 0.08)	0.18 (*P*= 0.08)
Gen, Gap; controlled for Geo	0.61 (*P*= 0.76)	−0.97 (*P* < 0.00)	0.20 (*P*= 0.07)

In the extended set of eight *D. arida* population samples, the correlation between genetic and geographic distances was positive, but not significant at the conventional alpha-level 0.05. With the effect of habitat discontinuity removed, the correlation was not significant either (Table 5). SAShA of the wider *D. arida* data yielded a 138.3 km mean distance between co-occurrences of alleles, which was not significantly different from the expected mean distance under panmixia, 141.3 km (*P*= 0.28).

### Discussion

In order to obtain a better understanding of the mechanisms and spatial scales of population-genetic connectivity in the marine environment, Kinlan and Gaines (2003) called for more attention to be paid to interactions between species that make up a community. Our study addressed the effects of interaction between a habitat-forming species of an intertidal community (*D. antarctica*) and grazing gastropods (*D. durvillaea*, *D. arida*), which differ in their reliance on and occupation of *D. antarctica* beds.

### Long-distance gene flow appears higher in macroalgal-associated species

Contrary to predictions, we did not find differences in the overall level of genetic structuring or in the magnitude of genetic distances between populations between the rock-associated *D. arida* versus kelp-associated *D. durvillaea* (Hypothesis 1). Both species apparently maintain high levels of gene flow across the study area. Nevertheless, in keeping with the predictions made at the outset of this study, we detected somewhat differing geographical patterns of connectivity (migrant numbers; correlations of geographic and genetic distances) between the two species (Hypothesis 2), suggesting that habitat associations can have some evolutionary consequences for intertidal marine taxa at the studied spatial scale. Specifically, populations of *D. durvillaea* were overall more connected across the major Canterbury Bight habitat discontinuity than were populations of *D. arida*. By necessity, *D. arida* must rely on larval dispersal alone to traverse this 180 km wide habitat gap, whereas *D. durvillaea* has the added potential to use rafting as its dispersal mechanism.

Our results indicate that the Canterbury Bight represents a weaker barrier to gene flow of *D. durvillaea* than of *D. arida*. The high migration rate estimates of *D. durvillaea* across the habitat discontinuity are consistent with recent studies of beach-cast bull-kelp in this region. In particular, a genetic survey showed that oceanographic transport of detached bull-kelp drifting across the Canterbury Bight (e.g., between study sites nos. 2–4 and 1) occurs on a regular basis (Collins et al. 2010),which suggests that higher migration rates of *D. durvillea* across the Canterbury Bight from sites nos. 1–2 could indeed be due to rafting events. The observed high levels of allelic diversity at site no. 4 at all studied loci suggest that gene flow across the Canterbury Bight is ongoing (as opposed to, e.g., a single colonization event). Rafting dispersal may be particularly important for local recolonization and population recovery in *D. durvillaea*, considering the susceptibility of *D. antarctica* to local population extinctions (Donald et al. 2011).

Based on our comparison of genetic connectivity of a kelp-associated and a rock-associated intertidal snail, we expect that species inhabiting strongly buoyant macroalgae that grow in environments affected by strong tidal and wind-driven currents are predisposed to gene flow by rafting dispersal. Rafting dispersal appears to have potential to enhance connectivity between populations that are not within easy reach of dispersal achieved by planktonic larvae. The absence of genetic isolation-by-distance/habitat gap pattern in the kelp-associated *D. durvillaea* and its presence in the rock-associated *D. arida*—albeit based on a very small number of datapoints and only one major habitat gap—implies that gene flow effects of rafting dispersal can be highly unpredictable; in particular, they are not necessarily dependent on geographic distance even under influence of a stable, unidirectional major current.

Our study focused on a kelp-associated snail that exploits only a very small part of the alga, the holdfast. We believe holdfast-associated species are particularly likely to survive rafting journeys, as the complex structure of holdfast cavities and tunnels of many large seaweed species provides good shelter from wave action. In spite of constituting only a small proportion of the total biomass of typical large macroalgae, holdfasts support far more epifaunal biodiversity than blades or stipes (Christie et al. 2003; Wlodarska–Kowalczuk et al. 2009). Therefore, the microevolutionary effects of rafting dispersal potentially affect a considerable part of marine intertidal biodiversity. Evidently, the scale at which rafting dispersal impacts population connectivity of a species is dependent on the species’ pelagic larval duration and maximum larval dispersal distance. Evolutionary effects of rafting dispersal are likely to be more pronounced in kelp-associated species that lack planktonic larvae, unlike *D. durvillaea*.

### Local gene flow seems higher in the rock-associated species

In line with the habitat preferences of the two snail species, our study suggests that *D. arida* has exchanged (on average) higher numbers of migrants across local scales in southern South Island (e.g., between sites nos. 1 and 3) where no sandy or gravel beaches longer than 8 km separate rocky intertidal habitats. As *D. arida* is abundant across this rocky region (with many additional populations that were not sampled for this study), there are enhanced opportunities for local gene flow. By contrast, local gene flow is presumably weaker in *D. durvillaea* because of the more limited bull-kelp habitat whose presence is more strictly dictated by the slope and wave exposure of the shore. The very large effective population size of *D. arida* (relative to the rarer and more restricted *D. durvillaea*; Spencer et al. 2009) may also partly account for the apparent lack of strong genetic differentiation among sampled populations, due to weaker effects of genetic drift (cf. Donald et al. 2011).

Despite the uniformly directional (south-to-north) flow of the Southland Current (Chiswell 2009), there is a possibility that variable rates of flow in this region could partly explain regional variation in gene flow estimates. For instance, the surface speeds recorded between sites nos. 1 and 2 have been estimated to be five times slower (typically below 10 cm/s) than in the region north of the Otago Peninsula between sites nos. 2 and 4 (Chiswell 1996). Thus, the relatively high speed of the Southland Current in the Canterbury Bight area could perhaps have contributed to the higher estimate of *D. arida* migrants between sites nos. 3 and 4. For example, at a steady speed of 50 cm/s, larval offspring from site no. 3 could potentially cross the habitat gap and reach site no. 4 within 5 days—a time interval that is roughly concordant with the life span of trochid veligers (Hickman 1992).

The validity of the inferences we have made on the patterns and strength of population-genetic structuring in the two *Diloma* species depends on the comparability of the microsatellite data between the species. Given that we inferred null alleles to be abundant in the data, it is important to consider whether they could have biased our between-species comparisons of patterns and strengths of population-genetic structuring, on one hand by disproportionately inflating all population-pairwise *F*_ST_ and *D*_C_ estimates (Chapuis and Estoup 2007) of one species relative to the other, and/or on the other hand by influencing different population-pairwise contrast in one species than in the other. Given that the estimated null allele frequencies were fairly similar between the species across populations and loci, we contend that the null allele correction methods of Chapuis and Estoup (2007)—that decrease *F*_ST_ and *D*_C_—were equally efficient for the two species and that null alleles have therefore not seriously biased our between-species comparisons. We also tested to see if the locus Ddu7 (whose amplification success differed greatly between the species) had affected the between-species comparisons and found out that it had not: for example, population-pairwise *D*_C_ distances from analyses that omitted Ddu7 correlated closely with those from the seven-locus data in both species (Pearson *r* > 0.98), and the absolute differences in the distances were small (*D. durvillaea*, mean Δ*D*_C_= 0.016, *SD*= 0.006; *D. arida*, mean Δ*D*_C_= 0.018, *SD*= 0.009).

Our study suggests that for intertidal snails with a planktonic larval dispersal stage, ecological association with a buoyant macroalga does not confer dramatic consequences to population-genetic structuring, at least on the geographic scale and genetic resolution achieved by our study. However, given that *D. durvillaea* has a very fragmented and sparse distribution compared to *D. arida*, it is remarkable that the two species exhibited comparable levels of genetic connectivity. Either the less common *D. durvillaea* populations must produce more gametes and larvae than *D. arida*, or their gene flow is enhanced by some other mechanism such as rafting of reproductively active adults. Genetic studies of species pairs with more balanced population distributions and population sizes are still needed to more robustly test the evolutionary significance of rafting dispersal for epifauna associated with buoyant macroalgae. To this end, we are preparing a comparison of population-genetic structuring in rock-associated versus kelp-associated chitons of the genus *Sypharochiton* along the eastern coast of New Zealand, using AFLP-markers. Importantly, this additional study will provide much needed phylogenetic replication for testing the dispersal-by-rafting hypothesis.

## Data Accessibility

DNA sequences: deposited in GenBank under accessions HQ434474-HQ434478, HQ434480, HQ434481.

Microsatellite primers: submitted to Molecular Ecology Resources Database as per Supplementary File S1.

Microsatellite data: Data deposited in the Dryad Repository: doi:10.5061/dryad.pb0dn.

Museum voucher specimens: deposited in The Museum of New Zealand Te Papa Tongarewa, Wellington.

## Supporting information

**Table S1.** Microsatellite primers for loci Ddu1-Ddu7, developed from clones of *Diloma durvillaea* DNA and used for genotyping *D. arida* and *D. durvillaea*.

## References

[b1] Bell JJ (2008). Similarity in connectivity patterns for two gastropod species lacking pelagic larvae. Mar. Ecol. Prog. Ser..

[b2] Cavalli-Sforza LL, Edwards AWF (1967). Phylogenetic analysis: models and estimation procedures. Evolution.

[b3] Chapuis MP, Estoup A (2007). Microsatellite null alleles and estimation of population differentiation. Mol. Biol. Evol..

[b4] Chiswell S (1996). Variability in the Southland Current, New Zealand. New Zeal. J. Mar. Freshwat. Res..

[b5] Chiswell S (2009). Colonisation and connectivity by intertidal limpets among New Zealand, Chatham and Sub-Antarctic Islands. II. Oceanographic connections. Mar. Ecol. Prog. Ser..

[b6] Christie H, Jorgensen NM, Norderhaug KM, Waage-Nielsen E (2003). Species distribution and habitat exploitation of fauna associated with kelp (*Laminaria hyperborea*) along the Norwegian coast. J. Mar. Biol. Assoc. UK..

[b7] Coleman MA, Brawley SH (2005). Are life history characteristics good predictors of genetic diversity and structure? A case study of the intertidal alga *Fucus spiralis* (Heterokontophyta; Phaeophyceae). J. Phycol..

[b8] Coleman MA, Kelaher BP (2009). Connectivity among fragmented populations of a habitat-forming alga, *Phyllospora comosa* (Phaeophyceae, Fucales) on an urbanised coast. Mar. Ecol. Prog. Ser..

[b9] Collins CJ, Fraser CI, Ashcroft A, Waters JM (2010). Asymmetric dispersal of southern bull-kelp (*Durvillaea antarctica*) adults in coastal New Zealand: testing an oceanographic hypothesis. Mol. Ecol..

[b10] Cowen RK, Sponaugle S (2009). Larval dispersal and marine population connectivity. Annu. Rev. Mar. Sci..

[b11] Donald KM, Keeney DB, Spencer HG (2011). Contrasting population makeup of two intertidal gastropod species that differ in dispersal opportunities. J. Exp. Mar. Biol. Ecol..

[b12] Donald KM, Kennedy M, Spencer HG (2005). Cladogenesis as the result of long-distance rafting events in South Pacific topshells (Gastropoda, Trochidae). Evolution.

[b13] Eikaas HS, Hemmingsen MA (2006). A GIS approach to model sediment reduction susceptibility of mixed sand and gravel beaches. Environ. Manage..

[b14] Fraser CI, Thiel M, Spencer HG, Waters JM (2010). Contemporary habitat discontinuity and historic glacial ice drive genetic divergence in Chilean kelp. BMC Evol. Biol..

[b15] Fraser CI, Nikula R, Waters JM (2011). Oceanic rafting by a coastal community. Proc. Roy. Soc. Lond. Ser. B Biol. Sci..

[b16] Gerlach G, Jueterbock A, Kraemer P, Deppermann J, Harmand P (2010). Calculations of population differentiation based on G(ST) and D: forget G(ST) but not all of statistics!. Mol. Ecol..

[b17] Glenn TC, Schable NA, Zimmer EA, Roalson E (2005). Isolating microsatellite DNA loci. Molecular evolution: producing the biochemical data, Part B.

[b18] Grange KR (1976). Rough water as a spawning stimulus in some trochid and turbinid gastropods. New Zeal. J. Mar. Freshw. Res..

[b19] Hickman CS (1992). Reproduction and development of trochacean gastropods. Veliger.

[b20] Jensen JL, Bohonak AJ, Kelley ST (2005). Isolation by distance, web service. BMC Genet..

[b21] Jost L (2008). G(ST) and its relatives do not measure differentiation. Mol. Ecol..

[b22] Kelly RP, Oliver TA, Sivasundar A, Palumbi SR (2010). A method for detecting population genetic structure in diverse, high gene-flow species. J. Hered..

[b23] Kinlan BP, Gaines SD (2003). Propagule dispersal in marine and terrestrial environments: a community perspective. Ecology.

[b24] Leese F, Agrawal S, Held C (2010). Long-distance island hopping without dispersal stages: transportation across major zoogeographic barriers in a Southern Ocean isopod. Naturwissenschaften.

[b25] McCormick TB, Buckley LM, Brogan J, Perry LM (2008). Drift macroalgae as a potential dispersal mechanism for the white abalone *Haliotis sorenseni*. Mar. Ecol. Prog. Ser..

[b26] Meirmans PG, Hedrick PW (2011). Assessing population structure: F-ST and related measures. Mol. Ecol. Res..

[b27] Muhlin JF, Engel CR, Stessel R, Weatherbee RA, Brawley SH (2008). The influence of coastal topography, circulation patterns, and rafting in structuring populations of an intertidal alga. Mol. Ecol..

[b28] Nikula R, Fraser CI, Spencer HG, Waters JM (2010). Circumpolar dispersal by rafting in two subantarctic kelp-dwelling crustaceans. Mar. Ecol. Prog. Ser..

[b29] Ota T (1993). DISPAN: genetic distance and phylogenetic analysis.

[b30] Peakall R, Smouse PE (2006). GENALEX 6: genetic analysis in Excel. Population genetic software for teaching and research. Mol. Ecol. Notes.

[b31] Perrin C, Roy MS (2000). Rapid and efficient identification of microsatellite loci from the sea urchin. *Evechinus chloroticus*. Mol. Ecol..

[b32] Pritchard JK, Stephens M, Donnelly P (2000). Inference of population structure using multilocus genotype data. Genetics.

[b33] Raven PH, Evert RF, Eichhorn SE (2005). Biology of plants.

[b34] R Development Core Team (2010). R: a language and environment for statistical computing.

[b35] Spencer HG, Marshall BA, Waters JM (2009). Systematics and phylogeny of a new cryptic species of *Diloma* Philippi (Mollusca: Gastropoda: Trochidae) from a novel habitat, the bull kelp holdfast communities of southern New Zealand. Invertebr. Syst..

[b36] Underwood AJ (1974). Reproductive cycles and geographical distribution of some common Eastern Australian prosobranchs (Mollusca-Gastropoda). Aust. J. Mar. Freshwat. Res..

[b37] Untergasser A, Nijveen H, Rao X, Bisseling T, Geurts R, Leunissen JA (2007). Primer3Plus, an enhanced web interface to Primer3. Nucleic Acids Res..

[b38] Van Oosterhout C, Hutchinson WF, Wills DPM, Shipley P (2004). MICRO-CHECKER: software for identifying and correcting genotyping errors in microsatellite data. Mol. Ecol. Notes.

[b39] Walsh PS, Metzger DA, Higuchi R (1991). Chelex-100 as a medium for simple extraction of DNA for PCR-based typing from forensic material. Biotechniques.

[b40] Weir BS (1996). Genetic data analysis II.

[b41] Wlodarska-Kowalczuk M, Kuklinski P, Ronowicz M, Legezynska J, Gromisz S (2009). Assessing species richness of macrofauna associated with macroalgae in Arctic kelp forests (Hornsund, Svalbard). Polar Biol..

[b42] Zavodna M, Sandland GJ, Minchella DJ (2008). Effects of intermediate host genetic background on parasite transmission dynamics: a case study using *Schistosoma mansoni*. Exp. Parasitol..

