# Supplementary material for: Comparison of population-genetic structuring in congeneric kelp- versus rock-associated snails: a test of a dispersal-by-rafting hypothesis
Source: Ecol Evol. 2011 Oct;1(2):169–80. doi: 10.1002/ece3.16 (PMC3287294; doi:10.1002/ece3.16)
Supplement: Supplementary file 1 [file ece30001-0169-SD1.pdf]

Supplementary File S1 Microsatellite primers for loci Ddu1-Ddu7, developed from clones of *Diloma durvillaea* DNA and used for genotyping *D. arida* and *D. durvillaea*. The used number of PCR-cycles (X), PCR-step duration (X), and annealing temperature (Z) are listed for each marker, along with the acrylamide concentration of the electrophoresis gel used to separate the amplified fragments and the mean observed (H_O) and expected (H_E) heterozygosities for each locus over sample localities #1-4 in each of the species (shaded: *D. durvillaea*, unshaded: *D. arida*; not corrected for null alleles). The generic PCR-cycling conditions used for amplifying the loci were: initial denaturation of 4 min at 94 °C, followed by X cycles of Y s at 94 °C, Y s at Z °C and Y s at 72 °C, and ending with a final extension step of 10 min at 72 °C.

Locus	Primer sequences 5'-3'	Repeats (in the sequenced clone between primer regions)<br>Genbank Accession of the cloned sequence	X	Y	Z	Gel	H_O	H_E
**Ddu1**	F:ACAAGTTGCCGAGACCAGTT	$(AC)_4$…$(ATT)_2$	28	15 s	50	10 %	0.35	0.65
	R:CGGTTCACATTAAATTCCTTC	HQ434474					0.27	0.51
**Ddu2**	F:TGCGCATAATAAATACTCCATT	$ACTA(ACT)_3T(CTA)_{15}T(TAC)_4T(TAG)_4G(CTA)_6G(ATT)_2T(ATT)_2$	25	25 s	50	8 %	0.47	0.94
	R:TGGCAGCAGTAATTGTAATAGTAGC	HQ434475					0.67	0.93
**Ddu3**	F:CGCCAGGAGTTGCAATTATC	$(AT)_2$…$(CA)_3(GA)_2$…$(CA)_3$…$(GA)_2$…$(TA)_2$…$(GA)_2$	28	20 s	49	8 %	0.30	0.42
	R:CAGGTGTCCACAGAAATGCT	HQ434476					0.13	0.32
**Ddu4**	F:GCTCCATGAGGTCTAAATTGG	$(AAC)_2(AT)_2$ $(CTT)_2$ …$(TAC)_7$… $(CTA)_{12}(CCA)_2$… $(ACT)_4$…$(TA)_2$	25	15 s	50	8 %	0.68	0.93
	R:AGGATCAGTTTGGTTTCATCAG	HQ434477					0.53	0.93
**Ddu5**	F:TCGTCGTCTGAGCATACTTCC	$(AAG)_{11}$	28	15 s	50	10 %	0.69	0.83
	R:GTGGCCTTTGATACCGCATA	HQ434478					0.44	0.71
**Ddu6**	F:ATAACGGCCGTCGGAGA	$(TGT)_4A(GTA)_4$…$(TTGA)_2(TC)_2$…$(CAT)_2$	25	25 s	48	9 %	0.13	0.21
	R:CAACGCAGTCAGAAACACAGA	HQ434480					0.57	0.66
**Ddu7**	F:GTCAACTTTACTATATGGAGAAAACC	$(AC)_2(CAC)_9(CAA)_{16}A_2(CAA)_3(CAG)_3$…$(ATT)_2$…$(AT)_2$	25	25 s	48	8 %	0.53	0.93
	R:TCAGTGTTATTAGATAACGTTTCCTGT	HQ434481					0.23	0.69
